# Divine thus good, devilish thus bad? Folk linguistic perceptions about plants and their characteristics in Polish folklore

**DOI:** 10.1186/s13002-025-00787-z

**Published:** 2025-07-25

**Authors:** Olga Kielak

**Affiliations:** Faculty of Languages, Literatures and Cultures, Maria Curie-Skłodowska University in Lublin, pl. Marii Curie-Skłodowskiej 4A, 20-031 Lublin, Poland

**Keywords:** Ethnolinguistics, Ethnobotany, Local plant names, Devilish plants, Divine plants

## Abstract

**Introduction:**

According to folklore, some plants are created by divine beings and holy persons, while others appear on earth through demonic intervention. It is commonly believed that plants of divine origin are “good” plants, useful to humans, while plants of devilish origin are “bad” and not useful.

**Aim of the study:**

This article analyses folk beliefs regarding the origins of selected plants, identifies which of them are considered to have a divine origin and which a demonic one, and examines whether the perceived divine or demonic origin of a plant influences its usefulness or harmfulness to humans.

**Methods:**

This article first compares folk beliefs regarding the origins of selected plants, identifies their divine and demonic origins, and then evaluates the characteristics of these plants (edible/inedible, desirable/undesirable in cultivation, used in folk medicine, used in rituals, blessed throughout the year, used in apotropaic practices, associated with the devil/used in black magic). The aim is to determine whether there are any correlations between these characteristics and the plants’ divine or demonic origins.

**Results and discussion:**

The analyses carried out have shown that a given plant’s divine or devilish provenance does not determine its usefulness or lack thereof, because in popular folkloristic imagery about plants we can find many characteristics that “escape” the sharp division into “good” and “bad” plants. Plants whose origin in folk imagery is associated with the activity of divine agents are edible plants, desirable to man, commonly used in (annual and family) rituals and in folk medicine, while plants associated with the devil are plants that are often poisonous, harmful, dangerous, stinging and prickly, undesirable as crops and classified as weeds, representing a dwelling place for forces hostile to man on the one hand, yet used in an apotropaic capacity on the other. At the same time, “devilish” plants were sometimes eaten as famine food, blessed and used in folk medicine, while “divine” plants, treated as weeds, were considered the abode of demons and used in black magic.

**Conclusion:**

The study shows that the divine or devilish provenance of plants can be interpreted as information about the source of a plant’s power—either divine or devilish. The article provides new insights for research on the perception of plants in Polish folk culture and also helps to promote Polish ethnolinguistic studies within the international academic discourse.

**Supplementary Information:**

The online version contains supplementary material available at 10.1186/s13002-025-00787-z.

## Introduction

In an ethnolinguistic description of the plant world reconstructed by means of the cognitive definition (for the application of this definition to the reconstruction of the plant world, see [1, 2]), taking into account the knowledge of plants established in language and in extralinguistic reality, we can find information related to, inter alia, their nomenclature, appearance and properties, time of flowering and harvesting, and place of growth; the practical, ritual, magical and medicinal use of particular plants and their symbolism—that is, as far as the collected documentation allows since the semantic subcategories with their actual content are not mechanically inscribed by the authors into the definitions of individual entries. Instead, they are reconstructed for each described plant to highlight the particular characteristics embedded within the analysed material from the perspective of the bearers and participants of the studied culture [3].

Additionally, the cognitive definitions of plants often include a facet related to their origin, which includes information about what the plant is derived from or how it came to be. When asking about origins, ethnolinguists are not only interested in the origin of the plant in the literal sense (understood as the country of origin of the plant in question), but also in mythical origins that arise from folk imagery. In the aforementioned folk imagery, some plants are believed to have been created by divine entities and holy persons, while others appear on earth through demonic beings.

According to folklorists and ethnographers, the “mythical” origin of individual plants is a consequence of the characteristics of those plants observed by village inhabitants, and folk etiological tales explain why some plants are considered “good,” useful to humans, while others are deemed “bad” and useless [4–11]. The question that arises is: do all plants originating from God (or more broadly, from divine beings) truly qualify as “good” (edible, beneficial to humans, with medicinal properties), while those stemming from the devil are “bad” (inedible, harmful, undesirable in cultivation, etc.)? I hypothesize that the divine or demonic origin of a plant does not determine its usefulness or uselessness for humans.

To verify this hypothesis, I will first compile folk narratives about the origins of selected plants in the article, determining their origin (divine or demonic), and then I will refer to the practical, ritual, magical, and medicinal uses of the plants mentioned.

In attempting to reconstruct the origins of particular plants, it is impossible to overlook their folk names that include a diabolical component or a sacronym. It cannot be ruled out that these plant names are echoes of once-prevalent belief narratives concerning the origins of plants—in other words, legends and beliefs may serve as the foundation for the creation of plant names [12].

Folk names of plants have fascinated researchers for a long time—not only linguists, but also folklorists, ethnographers, and botanists. In Poland, since the 1880s, research on the history of plant names has been conducted by the botanist and folklorist Józef Rostafiński [13–15], in Germany—by the botanist Heinrich Marzell, who published the results of his research in the monumental dictionary of plant names in the German language [16–20], and in England—by the poet, writer, and botanist Geoffrey Grigson [21]. It was quickly noticed that folk plant names provide valuable source material, based on which one can reconstruct the characteristics of individual plants—their appearance, taste, or smell, practical, ritual, magical, and medicinal uses, as well as beliefs and convictions associated with the plants [see e.g. 2, 22, 23].

Researchers have also become interested in plant names “bearing the names of witches and devils” [24]. It has been hypothesized that certain plant species carry the names of witches and devils because they are perceived by people as “negative” (toxic, weedy, thorny). Dutch-British botanists analysing this issue have shown that in the languages of Northwestern Europe and Germanic languages (including Dutch, English, Frisian, German, Norwegian, and Swedish), the plants onomasiologically associated with witch and devil are classified as weeds, most often toxic (thus the name may have acted as a warning against the plants’ harmful properties), while at the same time being used to protect against evil (hence the conclusion that the names may also have referred to apotropaic uses of these plants). The aforementioned botanists focused on analysing the names and assessing the species characteristics of the plants, without considering folk tales about their origins. However, these stories, it seems, would have confirmed and enriched their arguments. Furthermore, it is possible that folk phytonyms containing elements associated with the devil or sacred symbolism reflect past beliefs related to the plants’ origins. This would thus open the way for integrated ethnolinguistic and ethnobotanical analyses, where linguistic, cultural, and botanical knowledge are treated equally.

## Methods and sources

Documentary material on the basis of which an ethnolinguist can reconstruct the origin of plants is provided by the already mentioned folk tales about the origin of the world (including the origin of the world of plants and animals), which in Polish folklore are called “aetiological fables” [11], “aetiological legends” [25], “aetiological tales” [26]. Aetiological contents depicting the genesis of a phenomenon also appear in other genres of folklore, including proverbs, folk requests, songs or legends [11]. They are also present in ethnographic records—sometimes the information about the origin of a plant is recorded by an ethnographer only in the form of a laconic “mention of a belief”, which, nevertheless, confirms the occurrence of a particular imagery in a given area. Therefore, this article analyses Polish folk aetiological legends and ethnographic records which mention the origin of selected plants. It does not include numerous stories explaining the particular properties of plants, which were contributed to by divine persons or demonic figures (e.g. the juice of swallowwort has a red colour because—according to a folk legend—the swallow, taking pity on Christ suffering on the cross, drove away with the plant brought in its beak the flies that annoyed the dying Jesus and the plant was thus sprinkled with Christ’s blood, see [27]; St. John’s wort has spots on its leaves because it arose from thorns thrown by the devil during an unsuccessful pursuit of a certain girl, see [28]). The analyses also exclude legends in which divine beings contribute to the creation of the plant in an indirect way, e.g. texts according to which horseradish (*Cochlearia armoracia*) was supposed to have arisen from a nail that a gypsy stole to prevent the Lord Jesus from being nailed to the cross [29].

In addition to ethnographic records, which mention the origin of selected plants, the conducted analyses also employ contexts that make it possible to reconstruct representations of particular plants (thus bringing us closer to an answer to the question posed earlier). Folklore data (aetiological legends) and ethnographic data are analysed together with lexicographical data, as is customary in the work of Lublin ethnolinguists. However, the analyses presented here are based only on those folk names of plants that may reflect the beliefs about the origin of the plant that were once present in Polish folklore. The folklore texts and ethnographic contexts constituting the basis for the material come from ethnographic sources published at the turn of the nineteenth and twentieth centuries (monographs and articles published in ethnographic journals, including “Zbiór Wiadomości do Antropologii Krajowej”, “Materiały Antropologiczno-Archeologiczne i Etnograficzne”, “Wisła” or “Lud”). The contexts were excerpted based on the list of sources adopted by the ethnolinguistic team in Lublin working on the *Dictionary of Folk Stereotypes and Symbols*. The folk plant names cited in the article that include a diabolical component or a sacronym were sourced from Polish dialect dictionaries. The geographical scope of the source materials I collected covers the entire ethnically Polish area; occasionally, I also include materials from Poles living in the Polish-Belarusian and Polish-Ukrainian borderlands, as well as the Vilnius region.

Initially, I intended to focus my analyses only on folk accounts related to the origin of shrubs and bushes, in line with the research area outlined in my research project. In the course of my work, however, it turned out that “divine” and “devilish” plants can also be found among vegetables and industrial plants, herbs and flowers, and the use of materials from other lexical fields “strengthens” the argument to verify the hypothesis mentioned at the beginning of the article. I found it much easier to reach for materials from other lexical fields by reading dictionary entries included in the volumes of *Słownik stereotypów i symboli ludowych* [*Dictionary of Folk Stereotypes and Symbols*], dedicated to plants. Owing to the fact that the authors of the cognitive definitions of plants included in the dictionary meticulously compiled references to facilitate the location of source contexts of particular interest to my analyses, I could treat individual plant entries as a kind of *datasets* or open research data. The dictionary entries of particular importance to this study include: grain [30], wheat [31], oats [32], buckwheat [33], pea [34], bean [35], turnip [36], tobacco [37], snuff [38], St. John’s wort [28], nettle [39], quaking grass [40], flower [41], primrose [42], pansy [43] or strawberry [44].

The problem I had to face was the botanical identification of plant species mentioned in the source contexts. In the extensive documentation I collected (the research data on which the article is based has been published as a dataset in the Zenodo repository: https://zenodo.org/records/15301758), information about the botanical identification of the species was often missing. In such cases, I attempted—whenever possible—to identify the species using a comparative analysis of local (folk) names, data on the plant’s usage, physical descriptions of the species, or information about the species’ distribution range [45]. The scientific names of the plants discussed in the article were updated based on data from the Flora of Poland database (https://www.atlas-roslin.pl/index.html) and the Plants of the World Online database (https://powo.science.kew.org).

## Results and discussion

According to the biblical account, God created the world in seven consecutive days: on the first day, he created day and night as well as heaven and earth; on the second day, he separated the waters from the sky; on the third day, he created land, seas and plants; on the fourth day—the sun, moon and stars; on the fifth day—fowl and fish; on the sixth day—animals and man; on the seventh day, he rested.

Only some of the folk accounts of the origin of plants are consistent with the biblical Book of Genesis. Divine origins are attributed primarily to grain [30], cf*. Zboże posiał Bóg, dlatego tak sie nawet nazywa* [lit. Grain was sown by God, hence its name; note: in Polish *zboże* (grain) and *Bóg* (God) appear to have similar morphological basis] [46], grain is said to be a gift from God [47]; according to the proverb: *Zboże z bożej ręki wyszło, a zielisko to diabół po świecie roztrząchnoł* [Grain came out of God’s hand, and the weed was scattered around the world by the devil] [48]. In folk aetiological legends, grain grows abundantly where God touches the earth with his foot [7]; cf. the legend recorded in the Vilnius Region (in a variant of the legend, God is replaced by the Lord Jesus [7]).

Chodził Pan bóg żebrujący, grzesznych ludzi próbujący, tak mówili dla dzieci. Idzie Pan Bóg przez pole, a tam kto orze, biedny jaki człowieczek […], konik nędzny, w jakich łapciach obity czy chodakach. – Boże pomagaj, człowiecze! Co tu będziesz siać? – Będę siać żyto. Bożeńka da, to może i wyrośnie, będzie chleb. Poszedł [Bóg] dalej, orze drugi, obraca łubin, para koni, konie dobre. – Boże pomagaj! Co tu robisz? – Orzę na żyto. – Kiedy Pan Bóg – mówi Pan Bóg – da, to i wyrośnie. – Co mi tam Pan Bóg, ja i bez Pana Boga wiem, że mi tu wyrośnie żyto. Gdzie tego Pana Boga ślady byli, jak przeszedł przez tą rolę, tak takie żyto, kłosy takie ogromne jak krzaki. – O, ja nie wiedział, jakby ja wiedziawszy, toby ja był całe pole tym człowiekiem wyciągawszy. U tego przepadło, a u tego biednego tak zarodziło żyto, że on i całą rodzinę wykarmił i do państwa sprzedał, dla biednych dawał żyta [7].

The Lord God went begging, trying sinful people, that’s what they said to children. The Lord God is walking through a field, and there someone is ploughing, a poor little man [...], a miserable horse, in a kind of clogs or shoes. – God help you, man! What are you going to sow here? – I’m going to sow rye. If God permits, maybe it will grow, and there will be bread. He [God] went on, another one is ploughing, he turns a lupine, a pair of horses, good horses. – God help you! What are you doing here? – I am ploughing for rye. – Should the Lord God – says God – grant it, it will grow. – God forbid, I know I’ll grow rye here even without God. Where the footprints of the Lord God were, when he went through that field, the rye was so great, ears as huge as bushes. – Oh, I didn’t know, if I had known, I would have dragged that man through the whole field. This man’s crop failed, and the poor man’s field was so overgrown with rye that he fed his whole family and sold it to the state, giving rye to the poor.

Selected trees and shrubs (and their fruits) were also created by God. For instance, according to folk legend: *dęby, buki, sosny, jodły, świerki i inne drzewa sam Bóg posiał podczas stworzenia świata* [oaks, beeches, pines, firs, spruces and other trees were sown by God himself as he was creating the world] [49]; cf. also the colloquial account: *Pan Bóg stworzył wszysko, różne drzewa, różne krzewy, różne owoce, wszysko z woli Boży* [The Lord God created everything, various trees, various shrubs, various fruits, everything by the will of God] [50]. God also created flowers; the divine origin is attributed to, inter alia, *Primula veris*, called *klucze/kluczyki św. Piotra* [St. Peter’s keys] [42, 51], which, by the power of God was created from the keys to the gates of heaven, accidentally lost by St. Peter [52], or *Viola tricolor*, also called *brat z siostrą* [brother and sister] [53], *braciszki* [brothers] [43, 54], into which—according to folk legends—a brother and sister united by incestuous love [55] or brothers in love with each other (with their beauty) were turned by God, cf.:

A u nas pono w dawnych czasach zdarzyło się, że było dwu braci, co się bardzo kochali, i byli pono najładniejsi na świecie. Tak oni nic nie robili, jino się na siebie patrzyli i tak se gadali: „Jakiś ty śliczny, aniołowie w niebie nie są ładniejsi od ciebie?” Za to Pan Bóg ich skarał i zamienił w kwiatek, co się nazywa bratek [55].

And here, in the old days, there were two brothers who loved each other very much, and they were supposedly the prettiest in the world. They wouldn’t do anything but look at each other and say: “How beautiful you are, the angels in heaven aren’t prettier than you”. For this the Lord God rebuked them and turned them into a flower called pansy.

In general, however, folk depictions of the origin of plants differ significantly from biblical accounts of the creation of the world. In many peasant accounts, the world, including the world of plants, is sometimes the work not only of God, but also of the devil [5, 6]. Sometimes individual elements of reality gain their final shape only owing to the interaction of these two beings.

**A dualistic cosmogony** appears in folk legends about the origin of *Avena sativa* [32], *Fagopyrum esculentum*—called *hreczka* [33], *Brassica rapa* [36], thistle [56] and *Urtica dioica* / *Urtica urens* [39]. According to the aetiological legends, it was God who allocated some of the aforementioned plants to the devil for cultivation: oats and buckwheat [57] or, in the variants of the legends, oats and turnips [58], but after the intervention of St. Michael or St. Martin, thistle and nettle [57] or thistle and burr [58], i.e. the fruit of *Arctium*, fell to the devil, cf.:

Pan Bóg, kiedy tworzył świat rozdawał i rozdawał różne nasiona: ludziom, zwierzętom, ptakom i djabłu tez kinął kilka ziarnek owsa i hrecki, ale św. Michał rzekł: Panie, i to ludziom się przyda, a diabłu dość będzie ostu i pokrzywy. – To odbierz-ze mu tamte, a daj te, rozkazał Bóg. Anioł puścił się w lot za diabłem i zdybał go dzieś na rozstaju dróg, jak wrzescał bez ustanku: „Mój owiec, moja hrecka, moj owies, moja hrecka!” – Co plecies! Przebił mu bozy Anioł. – A bodajcie, zapomniałem – To ja ci przypomnę: twój oset, twoja pokrzywa. – A, dziękuję – i z ten porwał się diabeł i znów wrzescał: „Mój oset, moja pokrzywa!” [57].

The Lord God, when He created the world, gave out and distributed various seeds: to people, animals, birds. He also gave a few grains of oats and buckwheat to the devil, but St. Michael said: Lord, these are good for people too, the devil will be just fine with thistles and nettles. – So take those away from him and give him these, God commanded. The angel flew after the devil and caught up with him at the crossroads as the latter screamed incessantly: “My oats, my buckwheat, my oats, my buckwheat!” – What you’re saying! The angel of God stopped him. – I seem to have forgotten – So I’ll remind you: yours is thistle and nettle. – Oh, thank you – and with that the devil carried on and shrieked again: “My thistle, my nettle!”.

In other aetiological legends, thistle [59, 60], quite accurately called *diabli łoset* [devil’s thistle] in Polish dialect [61], *diable nasienie* [62] and *diabelskie nasienie* [devil’s seed] [57] or *ziele czartowskie* [devil’s weed] [63], was created by the devil—either with God’s permission, cf. *Oset stworzył diabeł, gdy P. Bóg stworzywszy rośliny, jemu także coś stworzyć pozwolił* [Thistle was created by the devil, when God, having created plants, also allowed the devil to create something] [55], or without his participation, cf.: *oset zasiał diabeł na złość Panu Bogu, a ludziom na ciężkie utrapienie i od tego czasu rośnie on wszędzie,* (…) *mimo że nieustannie go tępią ludziska* [Thistle was sown by the devil to spite the Lord God and to afflict people, and since then it has been growing everywhere, (…) even though people have been constantly exterminating it] [64].

While in the case of the nettle, also called *diabelskie nasienie* [devil’s seed] [57], we are able to determine the species, in Poland there are two native species of nettle: *Urtica dioica* and *Urtica urens*, in the case of the thistle—on the basis of the collected material—we are unable to determine which specific plant is referred to in folklore texts and ethnographic records. As Katarzyna Prorok, the author of the entry *oset* [thistle], which will be published in Volume 8 of *Słownik stereotypów i symbolów ludowych* [56], argues both in dialects and in colloquial Polish, the name *oset* [thistle] is ambiguous and polysemous. It refers not only to the plants which botany classifies as belonging to the *Carduus* genus, but also to other prickly plants such as *Cirsium arvense*, *Silybum marianum* or *Onopordum acanthium* [65, cf. also 66]. In the case of the contexts used in my analyses, ethnographers did not specify which thistle in particular is meant, calling different plants thistles.

Dualism also appears in legends that speak of the creation of bilberries (*Vaccinium myrtillus*) and cowberries (*Vaccinium vitis-idaea*). In one legend, God allowed the devil to create bilberries (*Vaccinium myrtillus*), which the devil filled with poison to harm humans. The berries were only made edible by God, who *zamienił tę truciznę w nieszkodliwą słodycz i na każdej jagodzie czarny krzyżyk zrobił dla znaku* [turned this poison into a harmless sweetness and made a sign of black cross on each berry] [67]. In another legend, the Lord Jesus, walking around the world, created sweet black berries (this probably refers to bilberries, *Vaccinium myrtillus*), while the devil, watching him, created berries *czerwoniuśkie jak błyski ognia, na krzewinkach o twardych liściach* [as red as flashes of fire, on shrubs with hard leaves] (cowberries, *Vaccinium vitis-idaea*). The poisonous berries created by the devil only became good after the intervention of the Lord Jesus, who *położył na nich znak krzyża i odtąd borówki, co po borach rosną, służą ku pożytkowi człowieka* [put the sign of the cross on them, and from then on the berries, which grow in the forests, serve for the benefit of man] [64].

Alongside legends echoing a dualistic cosmogony, we can also find accounts in which a **single creator is responsible for the act of creation**—in folk legends, this role is played by the Lord Jesus, the Virgin Mary and the devil.

In some folk legends, the Lord Jesus is portrayed as the creator of grain (in other variants, God appears in the place of the Lord Jesus, as mentioned earlier)—this grows luxuriantly in the field where the Lord Jesus walked [7], or the creator of *Triticum aestivum*—which *rodzi się* [is born] where the Lord Jesus *stąpnął nóżką* [stepped with his foot] [68].

In another folk legend, the Lord Jesus created flowers (to make the earth prettier) and told them where to grow [41], cf.: *Ty rozo jidz do łogrodu ji nad drogi. Ty niksiecie (kaczeniec) na wody i porowy*. (…) *Ty sanku (sasanka) do boru, ła ty niezapominajko tu, ty wrzosie – tu, ji tak po koleji do łostatniego* [You, rose, go to the garden over the roads. You, marsh marigold, to the ditches by the water. (…) You, pasqueflower, to the forest. And you, forget-me-not—here; you, heather—here; and so on, one after another, until the last one] [69].

The origin of *Viburnum opulus* is linked to the action of the Lord Jesus—in a legend recorded near Kielce, the Lord Jesus took pity on the beautiful woman called *Kalina* [Viburnum], devoid of offspring*,* and created *krzewinę z jagodami czerwoniuśkiemi, podobnemi do jéj twarzyczki i nazwał ją kaliną na wszystkie wieki* [a shrub with red berries similar to her face, and called it viburnum for all eternity] [70].

In folk accounts and aetiological legends, the origin of certain plants is also linked to the last hours of Jesus Christ’s life. The plant that was said to have arisen from the blood of Jesus [71] is *Hypericum perforatum*. In Poland, such a belief was recorded in the Tarnów region, but legends stating that *Hypericum perforatum* grew under the cross of Christ and gained magical powers thanks to the Saviour’s blood dripping on it are popular in Russian folklore [72, cf. also 73]. It is possible that echoes of the aforementioned legends, once also present on Polish soil, are the Polish folk names for *Hypericum perforatum*: *krew Pana Jezusa* [74], *Pana Jezusowa krew* [75], *krewka Pana Jezusa* [Lord Jesus’ blood] [76]; or *chrystowe/Chrystusowe ślozki* [Christ’s tears] [74, cf. 28].

In the aetiological tales, some of the plants are created by the Virgin Mary who is considered the patroness of plants, her cult was linked to the annual vegetation cycle of plants [77]. In legends, the tears of the Virgin Mary weeping over starving people [55] or the tears shed by the Virgin Mary at the foot of the cross [78], gave rise to *Pisum sativum*, called in Polish dialects *łzy Matki Boskiej* [tears of the Mother of God] [55, 79], sometimes *łzy Pana Jezusa* [tears of the Lord Jesus] [80, cf. 34], cf:

Bardzo dawno temu panował na ziemi okropny głód, a był on karą za grzechy ludzi. Ludzie umiérali z głodu. Bóg nie dawał się żadną miarą ubłagać. Wtedy Matka Boska poczęła płakać i błagać; łzy jéj spadały na ziemię i przemieniały się w groch, który ludzie zbiérali i spożywali. Po kilku dniach wyczekiwania lepszéj przyszłości doczekali się ludzie przebaczenia bożego za wstawiennictwem Matki Boskiéj. Od tego czasu poczęli ludzie groch siać i uprawiać i nazwali go łzami Matki Boskiej [55].

A very long time ago there was a terrible famine on earth, and it was a punishment for the sins of the people. People were starving, but God could not be appeased. Then the Mother of God began to weep and beg; her tears fell to the ground and turned into peas, which the people gathered and ate. After several days of waiting for a better future, the people obtained God’s forgiveness through the intercession of the Mother of God. From then on, people began to sow and cultivate the peas and called them tears of the Virgin Mary.

In other variants of the referenced legend, *Phaseolus vulgaris* was formed from the tears of the Virgin Mary weeping over the misery of the starving people [81, cf. 35].

The tears of the Mother of God also gave rise to *Briza media*—“grass with tiny, heart-shaped spikelets, trembling with the slightest gust of wind”, referred to in Polish dialects as *łzy Matki Boskiej* [54] or *ślozki Matki Boskiej* [tears of the Mother of God], *serduszki Matki Boskiej* [hearts of the Mother of God] [51, cf. 40]. According to the folk tale:

Było to strasznie dawno. Tak jak my, żyła na świecie Maryja, Matka Boża. Ciężko pracowała i wychowywała swojego Syna – Jezusa. Szła raz od prania smugiem i tak nieszczęśliwie stąpnęła na kamień, że skręciła sobie nogę. Padła z wielkiego bólu na murawę i zaczęła rzewnie płakać. A łzy jej, które padały na rosnącą trawę, zamieniały się w drobniutkie serduszka. Odtąd trawa ta cięgiem dygocze jak w febrze, a ludzie nazywają ją trzęsichą albo łzami Matki Bożej [64].

It was a very long time ago. Just like us, Mary, the Mother of God, lived in the world. She was working hard and bringing up her Son, Jesus. One day, returning from laundry, she was walking down a narrow strip of meadow, she stepped so miserably on a stone that she twisted her leg. She fell in great pain on the turf and began to weep tearfully. Her tears, which fell on the growing grass, turned into tiny hearts. Since then, the grass has been trembling as if in a fever, and people call it quaking grass or the tears of the Mother of God.

Thanks to the Virgin Mary, strawberries (*Fragaria vesca* / *Fragaria viridis*) were also created. According to a legend recorded in the Podhale region, the Virgin Mary appeared to a poor girl named *Koziombka*, who, wanting to save her family from a devastating famine, offered Jesus her most precious possession—the round, blood-red beads she had once received from her mother. When she tore them up and they scattered on the ground, Mother of God turned them *w pachnące jakby miód a cyrwone jako krew jagódecki* [into beads that smelled like honey and were red as blood]. Everyone in the neighbourhood started to pick them afterwards, the famine ended in Podhale, and people called them *koziombki*, after the girl [82, cf. 44].

According to folk beliefs, the Virgin also bestowed the hazel with hazelnuts—as a reward for hiding the Holy Family from Herod’s pursuit [71]:

Maryja z Józefem i Dzieciątkiem uciekali przed żołnierzami Heroda i ukryli się pod osiką. Ta zaczęła ze strachu drżeć. Józef powiedział, żeby wyszli spod tego drzewa, gdyż ono ma w sobie szatana, że źle im życzy. Schowali się więc pod leszczynę, która tak opuściła gałęzie, że byli dobrze schowani. Dlatego potem Maryja powiedziała do leszczyny: „Za to, że nas uratowałaś, będziesz karmiła ludzi”. Od tej pory leszczyna się dobrze rozrasta, a osika drży [71].

Mary with Joseph and the Infant fled from Herod’s soldiers and hid under an aspen tree. This one began to tremble with fear. Joseph told them to come out from under that tree, for it had Satan in it and he wished them ill. So they hid under the hazel tree, which lowered its branches so that they were well hidden. Therefore Mary then said to the hazel tree: “For having saved us, you will feed the people”. Since then, the hazel tree has grown well and the aspen tree has trembled.

In folk accounts, the devil also performs the act of creation. He is considered to be the creator not only of the thistle (as mentioned earlier), but also of *Agrostemma githago*. It used to be said that *Pan Bóg nasiał zboże, a diabeł kąkol* [God sowed the grain and the devil sowed the corncockle] [83], in the Kociewie region it was believed that the devil sowed the corncockle in the grain at midnight [84], and in Kashubia it was said that *kąkol to djablå pszenica* [the corncockle is devil’s wheat] [75]. Echoes of beliefs about the devilish origin of the plant can be found in its folk names: *diabeł* [devil], *diabełek* [little devil], *diabła rogi* [devil’s horns] [54], *diabelski kwiat* [devil’s flower] [85], *szatański kwiatuszek* [Satan’s flower], *złyducha kwiatek* [evil spirit’s flower] [86], *roślina diabelska* [devil’s plant] [85], *diabelskie ziele* [devil’s weed] [51, cf. 87].

In Polish folk culture, the devilish origin is also attributed to *Sambucus nigra*, called *diabli bez* [devil’s elder] in the Lublin region [88]. The association of the bush with the evil element (not only with the devil, but also with the witch) is strongly established in folklore [cf. 2]. Historically, the bush was believed to have been planted by the devil [89], for which reason it is sometimes referred to as *diabelskie nasienie* [devil’s seed] [71].

In the imagination of Kashubians, tobacco (*Nicotiana tabacum*) also has devilish origins. According to regional fables, the devil sowed tobacco in a peasant’s field and, when he lost the bet, he left it to the peasant; to this day, tobacco growing in fields is called *diôbelsci zelësko* [devil’s weed] in Kashubian [90, cf. 37]. Kashubians also acknowledge the devil as the inventor of snuff, a finely ground and spiced tobacco intended to be ingested through the nose, cf. *tabakã ustanowił purtk* [75, cf. 38].

## Divine thus good, devilish thus bad?

The remainder of this article intends to examine whether in Polish plants created by God, Lord Jesus and Mother of God (often onomasiologically associated with divine persons) were perceived by people exclusively as “positive” (“good”, edible, useful to humans), while plants created by the devil (often onomasiologically associated with the devil) were consistently perceived as “negative” (“bad”, harmful and unusable).

In Table 1, I have compiled selected characteristics of plants in Polish folklore (edible/inedible, desirable/undesirable in cultivation, used in folk medicine, used in rituals, blessed during the year, used for apotropaic purposes, associated with the devil/used in black magic) in order to determine whether there are connections between these characteristics of the plants and their divine or demonic origins.

**Table 1:**
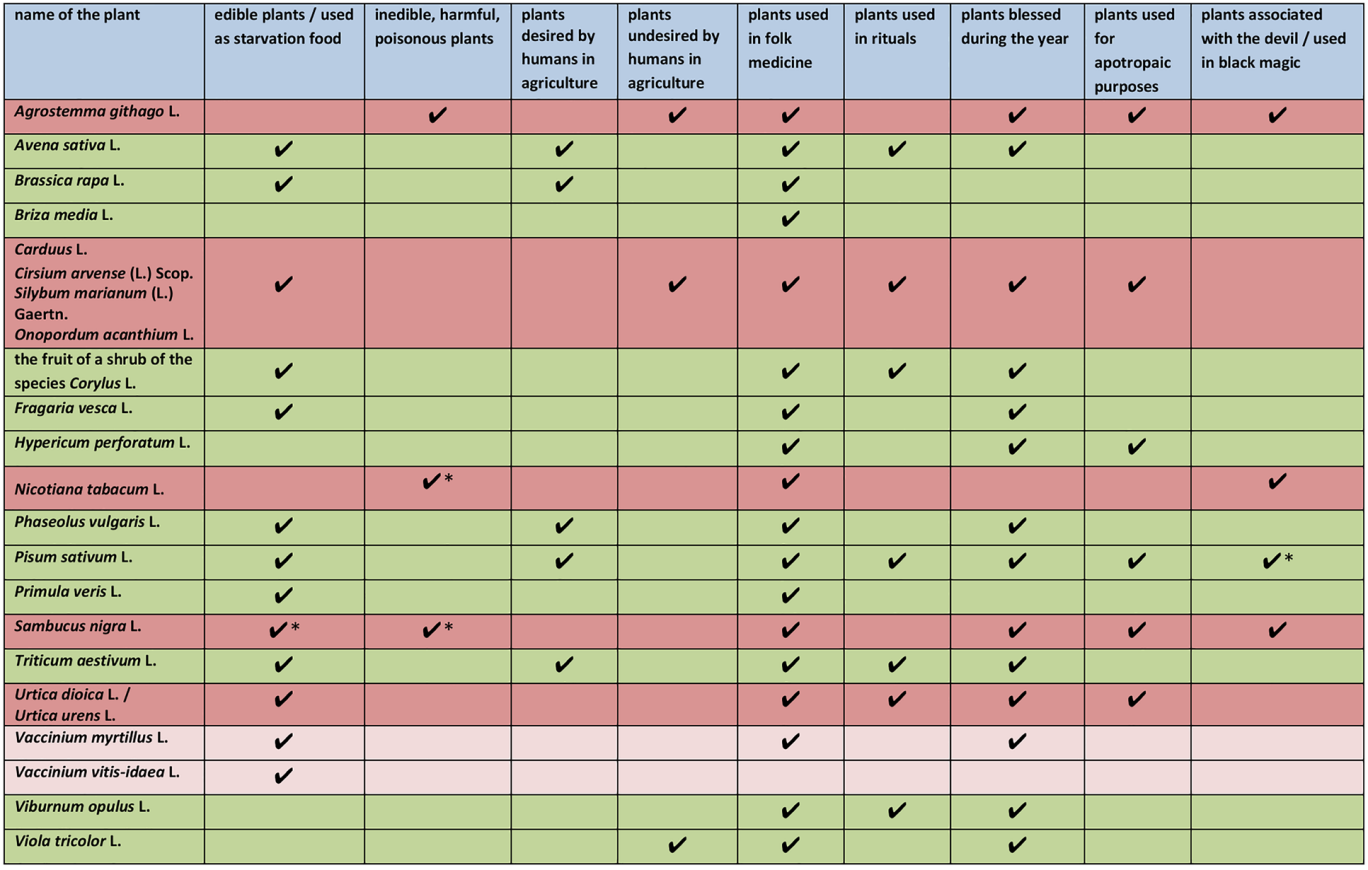
Selected characteristics of plants in Polish folklore

In Table 2 (which serves as supplementary material to Table 1), I have compiled selected contexts that provide readers with insight into how individual plants were used practically, ritually, magically, and medicinally by village inhabitants (for comprehensive cognitive definitions of the aforementioned plants [see, 28, 31–37, 39, 40, 42–44, 56, 122].

**Table 2:** Selected contexts illustrating the practical, ritual, magical, and medicinal use of plants

Divine plants	Devilish plants	
Edible plants	Used as starvation food	Inedible, harmful, poisonous plants
*Edible plants and inedible/harmful/poisonous plants*		
*Avena sativa* L. – oat grains are used to make oat flour and groats, from which everyday and ritual dishes and drinks are prepared [32] *Brassica rapa* L. – turnip was everyday food, eaten raw, cooked, dried, and smoked [36] fruit of the shrub of the *Corylus* L. – a supplement to the villagers’ everyday diet (common) *Fragaria vesca* L. – a supplement to the villagers’ everyday diet (common) *Phaseolus vulgaris* L. – a staple of the peasant diet (common) – also a festive dish, eaten on Christmas Eve and at weddings [35] *Pisum sativum* L. – a staple of the peasant diet – *to invite for peas* in old Polish meant ‘to invite for dinner’ [91] – a ritual dish, eaten on Christmas Eve and at weddings [34] *Primula veris* L. – used to make tea [92] *Triticum aestivum* L. – wheat grains are used to make wheat flour and groats, from which everyday and ritual dishes and drinks are prepared [31] *Vaccinium myrtillus* L. – a supplement to the villagers’ everyday diet (common) – edible only after divine intervention [67] *Vaccinium vitis-idaea* L. – a supplement to the villagers’ everyday diet (common)	*Carduus* L. *Cirsium arvense* (L.) Scop *Silybum marianum* (L.) Gaertn *Onopordum acanthium* L. – young thistle was famine food: in years of hunger, it was cooked like cabbage and eaten in early spring [64] – it was used to feed domestic animals [93] *Urtica dioica* L. – cf. *koprzywa*, *koprziwka*—a dish made from young nettles, prepared similarly to spinach [94] – used to feed domestic animals [93]	*Agrostemma githago* L. – poisonous [53] *Sambucus nigra* L. – raw fruits are inedible [95] – only suitable for consumption after heat processing (common) – used to make soup or preserves [96] *Nicotiana tabacum* L. – tobacco leaves, once dried and processed, are smoked, sniffed, or chewed [37] – harms people’s health [97] – causes cancer and asthma [37] – leaves bitterness and an unpleasant taste in the mouth [98]
Plants desired/undesired by humans in agriculture		
*Avena sativa* L. *Triticum aestivum* L. – there were traditional practices to ensure good yields. For example, on Ash Wednesday, farmers would once dance in the tavern “for the sake of oats and wheat”, believing that jumping high would help the crops grow tall [99] *Brassica rapa* L. – traditional abundance practices included, for example, smashing a pot on the sown turnip field so that the turnips would grow large and sweet [55] *Phaseolus vulgaris* L. – peasants knew when to sow and when not to sow beans, e.g. sow on a Monday to ensure a plentiful harvest [47]; do not sow on a day whose name contains the letter “r”, as the beans would become worm-infested and hard to cook [100] – there were traditional practices to ensure abundance. For example, to make the bean pods grow large and thick like a rolling pin, it was advised to thresh the seeds intended for sowing using a rolling pin [47] *Pisum sativum* L. – peasants knew when to sow and when not to sow peas, e.g. sow during the full moon so the pods are “full of seeds”; avoid sowing on weekdays that contain the letter “r” – Tuesdays, Wednesdays, and Thursdays – as worms would destroy the pods [47] – there were traditional practices to ensure abundance. For example, during Christmas Eve supper, the host would throw the first spoonful of the pea dish into the air, believing it would help peas grow tall [101] *Viola tricolor* L. – wild-growing pansy is categorized as a weed growing among cereal crops [102]	*Agrostemma githago* L. – categorized as a common cereal weed [53] – greatly contaminates the grain [51] – it is said to be “at odds with wheat” and dries it out [13] – removed from cultivated fields through ritual-magical practices: on Pentecost (Whitsun), boys would run around the fields with torches, shouting: *Uciekaj kąkoli, bo cię będę smolił! (Run away, corncockle, or I’ll scorch you!)* [103] *Carduus* L. *Cirsium arvense* (L.) Scop *Silybum marianum* (L.) Gaertn *Onopordum acanthium* L. – removed from fields using ritual-magical practices: on Christmas Day, household waste was swept from the house and thrown onto the field where thistles grew [55]; on St. Stephen’s Day, people would scatter oats on the field while saying: *Uciekaj, diable, z ostem, bo ja sieję święconym owsem* (*Devil, flee with the thistle, for I am sowing blessed oats!*) [104] *Urtica dioica* L. / *Urtica urens* L. – the word “nettle” could also mean “any weed” [105]	
Plants used in folk medicine		
*Avena sativa* L. – used in the treatment of high blood pressure; used in the treatment of respiratory diseases, mainly cough [106] *Brassica rapa* L. – used in the treatment of cough [107] – used in the treatment of toothache [55] *Briza media* L. – used in the treatment of heart diseases; used as a tonic [79] fruit of the shrub of the *Corylus* L. – used in the treatment of toothache [55] *Fragaria vesca* L. – used in the treatment of respiratory diseases; used in the treatment of skin diseases; used in the treatment of heart pain; used in the treatment of fever [79] *Hypericum perforatum* L. – used in the treatment of stomach pain and cough; used in the treatment of heart and kidney diseases [108] *Phaseolus vulgaris* L. – used in the treatment of kidney and urinary tract diseases [88] – used in the treatment of throat and lung diseases [88] *Pisum sativum* L. – used in the treatment of warts [109] – used in the treatment of corns [110] – used in the treatment of calluses [107] *Primula veris* L. – used in the treatment of lung diseases [75] – used in the treatment of toothache; used in the treatment of joint pain; used in the treatment of paralysis and trembling limbs [111] *Triticum aestivum* L. – used in the treatment of cough [57] – used in the treatment of erysipelas [112] *Vaccinium myrtillus* L. – used in the treatment of diarrhoea [51, 113] – used in the treatment of kidney diseases; used in the treatment of pinworms [113] *Viburnum opulus* L. – used in the treatment of cough [55] – used in the treatment of respiratory diseases [79] – used in the treatment of shortness of breath and asthma [88] *Viola tricolor* L. – used in the treatment of skin diseases [107] – used in the treatment of rashes in children [57] – used in the treatment of scabies [111]	*Agrostemma githago* L. – used in the treatment of headaches [114] – used in the treatment of colds in children [13] *Carduus* L. *Cirsium arvense* (L.) Scop *Silybum marianum* (L.) Gaertn *Onopordum acanthium* L. – used in the treatment of colic [115] – used in the treatment of sharp pains [88] – used in the treatment of stomach pain [108] – used in the treatment of burns and wounds [79] *Nicotiana tabacum* L. – used in the treatment of colds; used in the treatment of stomach pain [106] *Sambucus nigra* L. – used in the treatment of colds [88] – used in the treatment of cough [116] – used in the treatment of toothache [117] *Urtica dioica* L. – used in the treatment of rheumatism [118] – used in the treatment of colic [116] – used in the treatment of cough and shortness of breath [106]	
*Plants used in rituals*		
*Avena sativa* L. fruit of the shrub of the *Corylus* L – fertility stimulants, common practices of sprinkling oats and hazelnuts, for example, on St. Stephen’s Day (December 26), boys would throw oats and hazelnuts at girls after Mass [119] *Pisum sativum* L. *Triticum aestivum* L. – fertility stimulants, common practices of sprinkling peas and wheat, for example, newlyweds were showered with peas “so they would have as many children as there were grains” and with wheat “so that they would have abundance and plenty of bread” [120] *Viburnum opulus* L. – used in wedding rituals [121]	*Carduus* L. *Cirsium arvense* (L.) Scop *Silybum marianum* (L.) Gaertn *Onopordum acanthium* L. – during the initiation of a novice into the group of reapers, to test their endurance, their face would be rubbed with thistle [122], or they would be dragged with the largest and sharpest thistle across their bare skin [123] *Urtica dioica* / *Urtica urens* – during the initiation of a novice into the group of reapers, to test their endurance, a nettle would be placed around their neck [124]	
*Plants blessed during the year*		
*Avena sativa* L. – blessed with other herbs on the Feast of the Assumption of the Blessed Virgin Mary [125] Fruit of the shrub of the *Corylus* L. – blessed with other herbs on the Feast of the Assumption of the Blessed Virgin Mary [125] *Fragaria vesca* L. – blessed in wreaths during the octave of Corpus Christi [55] – blessed with other herbs on the Feast of the Assumption of the Blessed Virgin Mary [88] *Hypericum perforatum* L. – blessed in wreaths during the octave of Corpus Christi [106] – blessed with other herbs on the Feast of the Assumption of the Blessed Virgin Mary [55] *Phaseolus vulgaris* L. – blessed with other herbs on the Feast of the Assumption of the Blessed Virgin Mary [122] *Pisum sativum* L. – blessed with other herbs on the Feast of the Assumption of the Blessed Virgin Mary [79, 125] *Triticum aestivum* L. – blessed with other herbs on the Feast of the Assumption of the Blessed Virgin Mary [125, 126] *Vaccinium myrtillus* L. – blessed with other plants in the palm for Palm Sunday [88] *Viburnum opulus* L. – blessed in wreaths during the octave of Corpus Christi [125] – blessed with other herbs on the Feast of the Assumption of the Blessed Virgin Mary [125] *Viola tricolor* L. – blessed in wreaths during the octave of Corpus Christi [55]	*Agrostemma githago* L. – blessed in wreaths during the octave of Corpus Christi [88, 125] – blessed with other herbs on the Feast of the Assumption of the Blessed Virgin Mary [55] *Carduus* L. *Cirsium arvense* (L.) Scop *Silybum marianum* (L.) Gaertn *Onopordum acanthium* L. – blessed in wreaths during the octave of Corpus Christi [127] – blessed with other herbs on the Feast of the Assumption of the Blessed Virgin Mary [126] *Sambucus nigra* L. – blessed in wreaths during the octave of Corpus Christi [128] – blessed with other herbs on the Feast of the Assumption of the Blessed Virgin Mary [126] *Urtica dioica* L. / *Urtica urens* L. – blessed in wreaths during the octave of Corpus Christi [106, 125] – blessed with other herbs on the Feast of the Assumption of the Blessed Virgin Mary [108]	
*Plants used for apotropaic purposes*		
*Hypericum perforatum* L. – carried as a remedy against curses [55] *Pisum sativum* L. – pea decoction protected cows from charms [79]	*Agrostemma githago* L. – with the blessed flower of the corncockle, housewives would fumigate “bewitched” cows [129] *Carduus* L. *Cirsium arvense* (L.) Scop *Silybum marianum* (L.) Gaertn *Onopordum acanthium* L. – it was placed on the threshold to protect against witches [130] *Sambucus nigra* L. – twigs of black elder were placed in windows, doors, and farm buildings as protection against witches [71] *Urtica urens* L. – twigs of nettle were inserted into roofs [128], in windows [131] for protection against witches—spoiled cow’s milk that was bewitched by witches was strained through nettle to “repair” it [132]	
plants associated with the devil/used in black magic		
*Pisum sativum* L. – used in black magic – to provoke an argument [133], cast a spell [46], bring death to someone [134]	*Agrostemma githago* L. – attracts lightning [135] – it should not be picked, as this may lead to death by lightning and possession [84] – the devil often resides in the corncockle [84] *Sambucus nigra* L. – an untouchable shrub [136] – it is forbidden to dig it up [137], cut it [132] without risking unpleasant consequences, such as illness [138], paralysis, the withering of the hand [71], death [132] – the shrub is inhabited by numerous demonic beings: an evil spirit [71], the devil [138] *Nicotiana tabacum* L. – has a demonic origin [75] – an attribute of demonic and supernatural beings [37]	

Plants whose origin is associated in folk imagery with the activity of divine beings are **edible** plants—such as grain, which provided farmers with abundance and sustenance, simply called *bread* [30], constituting the basis of peasant diet—such as peas and beans—created by the Mother of God for people to have something to eat, or supplementing the poor villagers’ daily food—such as bilberries, strawberries or hazelnuts.

These are plants **desired by man**, as evidenced by numerous “harvest boosting” practices, e.g. at Christmas Eve supper the host eating peas would throw the first spoonful of the dish upwards so that the peas would grow tall [101]; on Ash Wednesday the hosts used to dance in the inn “for oats and wheat” in the belief that jumping up would influence the growth of the plants [99].

These are also plants **used in** (annual, family) **rituals** as fertility stimulants, ensuring fertility, prosperity and life-giving force. The old agrarian rituals at Christmas or wedding ceremonies were often accompanied by the practice of sprinkling peas, wheat, oats or hazelnuts. For instance, on St. Stephen’s Day (26 December), the boys would throw oats and hazelnuts at the girls after mass [119]; the bride and groom were sprinkled with peas “so that they would have as many offspring as there were peas” and wheat “so that they would not lack fertility and bread” [120].

The aforementioned plants were also used **in folk medicine**. For instance, dried quaking grass, whose spikelets are shaped like a heart, was used to treat heart diseases [79]; beans, which resemble human kidneys and whose flowers resemble the shape of a human throat [35], were used to treat kidney and urinary tract diseases as well as throat and lungs diseases [88]. An infusion of strawberry leaves was also used to treat respiratory diseases [79], viburnum fruit was used to treat cough [55] and other respiratory conditions [79], and primrose infusion was used to treat lung diseases [75]. Pea was used to treat skin diseases resembling pea seeds [34], including papules [109], warts [113], corns [107]. Pansy was also regarded as a cure for skin diseases [107]; the decoction of pansy was used, inter alia, for children’s rashes [57] or scabies [111]. Hazelnuts, on the other hand, were commonly used to treat toothache [55]—the underlying reason for the medicinal practice may have been a desire to lend the teeth the hardness of hazelnuts; they were also used prophylactically because, as was believed, “one must bite nuts to keep one’s teeth healthy” [139].

The image of plants linked in the aetiological accounts to the devil is slightly different. These are often **poisonous** plants, such as elder, whose raw fruit is inedible [95], and can only be eaten after heat treatment; also corncockle [83], or cowberries and bilberries, which, according to some accounts, only ceased to be poisonous after divine intervention; although tasty, **harmful**—like tobacco, which “spoils people’s health” [97], causes cancer and asthma [95], and leaves bitterness and distaste in the mouth [99, cf. 37]; **dangerous**—like corncockle, believed to attract lightning [135] and not to be plucked as one risks death from lightning and possession [84], or like the elder, an untouchable shrub [131], which cannot be dug up [137], cut down [132] without risking unpleasant consequences in the form of, inter alia, illness [138], paralysis [55], withering of the hand [71], death [132].

“Devilish” plants are usually **undesirable as crops** and **are counted as weeds**—such as corncockle, which was said to “make a great mess of grain” [51], thistle or nettle; interestingly, in the Kashubian region, *nettle* is a name used for any weed [105]. The aforementioned plants were also removed from fields by means of numerous ceremonial and magical practices, e.g. on Christmas Day rubbish was swept from the room into a field where the thistle grew [55] or on St. Stephen’s Day oats was spread over the field with the words: *Uciekaj, diable, z ostem, bo ja sieję święconym owsem!* [Devil, flee with the thistle, for I am sowing blessed oats!] [105, cf. 56]; at Pentecost, boys would go round the fields with torches, shouting: *Uciekaj kąkoli, bo cię będę smolił!* [Flee corncockle, for I will put you on fire!] [103] to prevent corncockle and other weeds as well as grain diseases [87].

The **place** where they grew is the **abode of forces hostile to man**—according to folk beliefs, the devil likes to dwell in the corncockle [84], while the elder bush is inhabited by numerous demonic beings: the evil spirit [71], the devil [138].

The plants created by the devil are also literally **“unpleasant”** for humans, and touching them is extremely unpleasant: they **sting**, e.g. the nettle mentioned in the riddle: *bez nóg, bez rąk, a ugryzie jako bąk* [without legs, without hands, but bites like a bug], or **prick**, e.g. the thistle in the riddle: *złota laseczka, na wierzchu pałeczka, kto jej sie dotknie, temu krew cieknie* [a golden stick, on top of a twig, whoever touches it, will bleed] [140]. The aforementioned properties of plants were used, inter alia, in initiation rites—during the admission of a novice to the ranks of reapers, in order to test their stamina, nettle or thistle was put around their neck [125, cf. 39].

In folk culture, plants associated with the “world of evil” could at the same time serve as “remedies against evil powers” [141], which is why some plants with devilish origins were also used **apotropaically**—sprigs of elder, nettle and thistle were spread on the thresholds of houses, stuck in windows and in the roof sheathing, put on the thatch, stuck in farm rooms (in the cowshed, stables) with the intention of protecting against the witch (and the devil) [71, 128, 131]; housewives used the blessed corncockle flower to incense “bewitched” cows [129], and spoiled cow’s milk was sieved through nettles in order to repair it [132].

Some of the “devilish” plants also had numerous uses in **folk medicine**. For instance, elder, called *bez lekarski* [medical elder] [96] or *bez apteczny* [apothecary elder] [79], was used to treat the common cold [88], cough [116] and toothache [117]; thistle, *kolkowe ziele* [*colicky weed*] [117], was used to treat colic [115] and stabbing pains, while nettle was believed to be “good for everything” [88] and used for rheumatic pains (Wisła 1903/250), colic—because “its leaves sting like needles” [116] as well as cough and shortness of breath [106].

Despite their devilish origin, thistle [127, 142], corncockle [55, 88], nettle [106, 108] or elder [89, 128] were blessed in garlands for Corpus Christi, and also on the day of Our Lady of the Herbs (15 August)—along with cereals, herbs, flowers, some vegetables and twigs of shrubs and trees believed to be of divine origin.

In the folk imagery of plants we can find more such characteristics that “escape” the sharp division into “good” and “bad” plants. Peas, which were created from the tears of the Virgin Mary, were the food not only for humans but also demons, and a field sown with peas was believed to be their abode [34]; they were also used in black magic—to stir up a quarrel [133], to cast a spell [46] or even to bring death on someone [134]. Pansies, which were created by the Lord God, are categorised both as herbs [55] (from the herbalist’s point of view) and as weeds [102] (from the farmer’s point of view). Some of the devilish plants, including thistle and nettle, were eaten as famine food [64], especially in spring and in times of crop failure [39]; they were fed to domestic animals [93]; meat was wrapped in nettles—to prevent it from spoiling and crumbling [108]. The elder planted by the devil was given the epithet *holy* in folk requests; diseases, such as toothache, were transferred to the bush, imploring: *Święty bzie, weź moje bolenie pod swoje zdrowe korzenie* [Holy elder, take my ailment under your healthy roots] [107].

## Conclusion

A people’s vision of the world, as Joanna Tomicka and Ryszard Tomicki wrote:

is an expression of the inexhaustible, universal aspiration to make sense of all phenomena, to find a formula to explain the origin of the world, of people and of all beings; it is an expression of the deep desire to understand the reality that surrounds man [4].

The analysis of the collected material shows that plants of divine origin (created by God, the Lord Jesus or the Virgin Mary) are edible plants, often forming the basis of peasant diet, desired by man (numerous practices were used to ensure good harvest), used in annual and family rituals (ensuring fertility, prosperity and life-giving force) and in folk medicine. However, there are some cracks and fissures in this undoubtedly positive image of “good” and “useful” plants—some of them are categorised as weed; others are sometimes eaten willingly by demons and the fields where they grow are the abode of unclean forces; they are used in black magic.

Plants of devilish origin are often poisonous, harmful, dangerous, usually undesirable for cultivation and classified as weeds, the dwelling place of forces hostile to man; “unpleasant” also in the literal sense (stinging, prickly); commonly used apotropaically and, interestingly, also in folk medicine. Despite their devilish origin, some of them are eaten by people (formerly they were famine food), blessed in garlands for Corpus Christi or in bouquets on the Day of Our Lady of the Herbs (15 August), and some are even called *holy*.

Consequently, one gets the impression that the divine or devilish provenance of a plant does not determine whether it is useful or not, and in the case of traditional culture, a simple, bipolar division into “good” and useful plants on the one hand and “bad” and unusable plants on the other hand, does not really work. All plants—both those of divine and of devilish origin—were used in many ways. It is therefore proposed that the divine or devilish provenance of a plant be interpreted as information about the source of the plant’s power—either divine or devilish.

The article provides new insights for research on the perception of plants in Polish folk culture and, as was also my intention, helps to popularize Polish ethnolinguistic studies within the international academic discourse. In the future, research could be undertaken to examine whether, in the folklore of other European countries, there exists a connection between folk aetiological tales about the origins of plants, the folk names of these plants, and their characteristics.

## Supplementary Information

Additional file1 (PDF 515 KB)

Additional file2 (PDF 680 KB)

## Data Availability

The datasets during and/or analysed during the current study are available from the corresponding author on reasonable request. The research data constituting the database on which the analyses are based have been published in the Zenodo repository: https://zenodo.org/records/15301758.
