# Supplementary material for: Divine thus good, devilish thus bad? Folk linguistic perceptions about plants and their characteristics in Polish folklore
Source: J Ethnobiol Ethnomed. 2025 Jul 25;21:53. doi: 10.1186/s13002-025-00787-z (PMC12291273; doi:10.1186/s13002-025-00787-z)
Supplement: Supplementary file 1 — Additional file1 (PDF 515 KB) [file 13002_2025_787_MOESM1_ESM.pdf]

**Table 1. Selected characteristics of plants in Polish folklore**

| name of the plant | edible plants / used as starvation food | inedible, harmful, poisonous plants | plants desired by humans in agriculture | plants undesired by humans in agriculture | plants used in folk medicine | plants used in rituals | plants blessed during the year | plants used for apotropaic purposes | plants associated with the devil / used in black magic |
|---|---|---|---|---|---|---|---|---|---|
| *Agrostemma githago* L. | | ✔ | | ✔ | ✔ | | ✔ | ✔ | ✔ |
| *Avena sativa* L. | ✔ | | ✔ | | ✔ | ✔ | ✔ | | |
| *Brassica rapa* L. | ✔ | | ✔ | | ✔ | | | | |
| *Briza media* L. | | | | | ✔ | | | | |
| *Carduus* L.<br>*Cirsium arvense* (L.) Scop.<br>*Silybum marianum* (L.) Gaertn.<br>*Onopordum acanthium* L. | ✔ | | | ✔ | ✔ | ✔ | ✔ | ✔ | |
| the fruit of a shrub of the species *Corylus* L. | ✔ | | | | ✔ | ✔ | ✔ | | |
| *Fragaria vesca* L. | ✔ | | | | ✔ | | ✔ | | |
| *Hypericum perforatum* L. | | | | | ✔ | | ✔ | ✔ | |
| *Nicotiana tabacum* L. | | ✔* | | | ✔ | | | | ✔ |
| *Phaseolus vulgaris* L. | ✔ | | ✔ | | ✔ | | ✔ | | |
| *Pisum sativum* L. | ✔ | | ✔ | | ✔ | ✔ | ✔ | ✔ | ✔* |
| *Primula veris* L. | ✔ | | | | ✔ | | | | |
| *Sambucus nigra* L. | ✔* | ✔* | | | ✔ | | ✔ | ✔ | ✔ |
| *Triticum aestivum* L. | ✔ | | ✔ | | ✔ | ✔ | ✔ | | |
| *Urtica dioica* L. /<br>*Urtica urens* L. | ✔ | | | | ✔ | ✔ | ✔ | ✔ | |
| *Vaccinium myrtillus* L. | ✔ | | | | ✔ | | ✔ | | |
| *Vaccinium vitis-idaea* L. | ✔ | | | | | | | | |
| *Viburnum opulus* L. | | | | | ✔ | ✔ | ✔ | | |
| *Viola tricolor* L. | | | | ✔ | ✔ | | ✔ | | |
