# Supplementary material for: Divine thus good, devilish thus bad? Folk linguistic perceptions about plants and their characteristics in Polish folklore
Source: J Ethnobiol Ethnomed. 2025 Jul 25;21:53. doi: 10.1186/s13002-025-00787-z (PMC12291273; doi:10.1186/s13002-025-00787-z)
Supplement: Supplementary file 2 — Additional file2 (PDF 680 KB) [file 13002_2025_787_MOESM2_ESM.pdf]

Table 2. Selected contexts illustrating the practical, ritual, magical, and medicinal use of plants

<table>
<tr><th>divine plants</th><th colspan="2">devilish plants</th></tr>
<tr><th colspan="3">edible plants and inedible/harmful/poisonous plants</th></tr>
<tr><th>edible plants</th><th>used as starvation food</th><th>inedible, harmful, poisonous plants</th></tr>
<tr>
<td>***Avena sativa* L.**
– oat grains are used to make oat flour and groats, from which everyday and ritual dishes and drinks are prepared [32]

***Brassica rapa* L.**
– turnip was everyday food, eaten raw, cooked, dried, and smoked [36]

fruit of the shrub of the *Corylus* L.
– a supplement to the villagers' everyday diet (common)

***Fragaria vesca* L.**
– a supplement to the villagers' everyday diet (common)

***Phaseolus vulgaris* L.**
– a staple of the peasant diet (common)
– also a festive dish, eaten on Christmas Eve and at weddings [35]

***Pisum sativum* L.**
– a staple of the peasant diet
– *to invite for peas* in old Polish meant 'to invite for dinner' [91]
– a ritual dish, eaten on Christmas Eve and at weddings [34]

Primula veris **L.**
– used to make tea [92]

Triticum *aestivum* **L.**
– wheat grains are used to make wheat flour and groats, from which everyday and ritual dishes and drinks are prepared [31]

***Vaccinium myrtillus* L.**
– a supplement to the villagers' everyday diet (common)
– edible only after divine intervention [93]

***Vaccinium vitis-idaea* L.**
– a supplement to the villagers' everyday diet (common)</td>
<td>***Carduus* L.**
***Cirsium arvense* (L.) Scop.**
***Silybum marianum* (L.) Gaertn.**
***Onopordum acanthium* L.**
– young thistle was famine food: in years of hunger, it was cooked like cabbage and eaten in early spring [64]
– it was used to feed domestic animals [94]

***Urtica dioica* L.**
– cf. *koprzywa*, *koprziwka* – a dish made from young nettles, prepared similarly to spinach [95]
– used to feed domestic animals [94]</td>
<td>***Agrostemma githago* L.**
– poisonous [53]

***Sambucus nigra* L.**
– raw fruits are inedible [96]
– only suitable for consumption after heat processing (common)
– used to make soup or preserves [97]

***Nicotiana tabacum* L.**
– tobacco leaves, once dried and processed, are smoked, sniffed, or chewed [37]
– harms people's health [98]
– causes cancer and astma [37]
– leaves bitterness and an unpleasant taste in the mouth [99]</td>
</tr>
<tr><th colspan="3">plants desired / undesired by humans in agriculture</th></tr>
<tr>
<td>***Avena sativa* L.**
Triticum *aestivum* **L.**
– there were traditional practices to ensure good yields. For example, on Ash Wednesday, farmers would once dance in the tavern "for the sake of oats and wheat", believing that jumping high would help the crops grow tall [100]

***Brassica rapa* L.**</td>
<td colspan="2">***Agrostemma githago* L.**
– categorized as a common cereal weed [53]
– greatly contaminates the grain [51]
– it is said to be "at odds with wheat" and dries it out [13]
– removed from cultivated fields through ritual-magical practices: on Pentecost (Whitsun), boys would run around the fields with torches, shouting: *Uciekaj kąkoli, bo cię będę smolił! (Run away, corncockle, or I'll scorch you!)* [104]</td>
</tr>
</table>

– traditional abundance practices included, for example, smashing a pot on the sown turnip field so that the turnips would grow large and sweet [55]<br><br>***Phaseolus vulgaris* L.**<br>– peasants knew when to sow and when not to sow beans, e.g. sow on a Monday to ensure a plentiful harvest [47]; do not sow on a day whose name contains the letter "r", as the beans would become worm-infested and hard to Cook [101]<br>– there were traditional practices to ensure abundance. For example, to make the bean pods grow large and thick like a rolling pin, it was advised to thresh the seeds intended for sowing using a rolling pin [47]<br><br>***Pisum sativum* L.**<br>– peasants knew when to sow and when not to sow peas, e.g. sow during the full moon so the pods are "full of seeds"; avoid sowing on weekdays that contain the letter "r" – Tuesdays, Wednesdays, and Thursdays – as worms would destroy the pods [47]<br>– there were traditional practices to ensure abundance. For example, during Christmas Eve supper, the host would throw the first spoonful of the pea dish into the air, believing it would help peas grow tall [102]<br><br>***Viola tricolor* L.**<br>– wild-growing pansy is categorized as a weed growing among cereal crops [103]	***Carduus* L.**<br>***Cirsium arvense* (L.) Scop.**<br>***Silybum marianum* (L.) Gaertn.**<br>***Onopordum acanthium* L.**<br>– removed from fields using ritual-magical practices: on Christmas Day, household waste was swept from the house and thrown onto the field where thistles grew [55]; on St. Stephen's Day, people would scatter oats on the field while saying: *Uciekaj, diable, z ostem, bo ja sieję święconym owsem* (*Devil, flee with the thistle, for I am sowing blessed oats!*) [105]<br><br>***Urtica dioica* L. / *Urtica urens* L.**<br>– the word "nettle" could also mean "any weed" [106]
**plants used in folk medicine**	
Avena sativa* L.**<br>– used in the treatment of high blood pressure; used in the treatment of respiratory diseases, mainly cough [107]<br><br>Brassica rapa* L.**<br>– used in the treatment of cough [108]<br>– used in the treatment of toothache [55]<br><br>***Briza media* L.**<br>– used in the treatment of heart diseases; used as a tonic [79]<br><br>**fruit of the shrub of the *Corylus* L.**<br>– used in the treatment of toothache [55]<br><br>***Fragaria vesca* L.**<br>– used in the treatment of respiratory diseases; used in the treatment of skin diseases; used in the treatment of heart pain; used in the treatment of fever [79]<br><br>***Hypericum perforatum* L.**<br>– used in the treatment of stomach pain and cough; used in the treatment of heart and kidney diseases [109]<br><br>***Phaseolus vulgaris* L.**<br>– used in the treatment of kidney and urinary tract diseases [88]<br>– used in the treatment of throat and lung diseases [88]<br><br>***Pisum sativum* L.**<br>– used in the treatment of warts [110]	***Agrostemma githago* L.**<br>– used in the treatment of headaches [115]<br>– used in the treatment of colds in children [13]<br><br>***Carduus* L.**<br>***Cirsium arvense* (L.) Scop.**<br>***Silybum marianum* (L.) Gaertn.**<br>***Onopordum acanthium* L.**<br>– used in the treatment of colic [116]<br>– used in the treatment of sharp pains [88]<br>– used in the treatment of stomach pain [109]<br>– used in the treatment of burns and wounds [79]<br><br>***Nicotiana tabacum* L.**<br>– used in the treatment of colds; used in the treatment of stomach pain [107]<br><br>***Sambucus nigra* L.**<br>– used in the treatment of colds [88]<br>– used in the treatment of cough [117]<br>– used in the treatment of toothache [118]<br><br>***Urtica dioica* L.**<br>– used in the treatment of rheumatism [119]<br>– used in the treatment of colic [117]<br>– used in the treatment of cough and shortness of breath [107]

– used in the treatment of corns [111]<br>– used in the treatment of calluses [108]<br><br>***Primula veris*** **L.**<br>– used in the treatment of lung diseases [75]<br>– used in the treatment of toothache; used in the treatment of joint pain; used in the treatment of paralysis and trembling limbs [112]<br><br>***Triticum aestivum*** **L.**<br>– used in the treatment of cough [57]<br>– used in the treatment of erysipelas [113]<br><br>***Vaccinium myrtillus*** **L.**<br>– used in the treatment of diarrhea [51, 114]<br>– used in the treatment of kidney diseases; used in the treatment of pinworms [114]<br><br>***Viburnum opulus*** **L.**<br>– used in the treatment of cough [55]<br>– used in the treatment of respiratory diseases [79]<br>– used in the treatment of shortness of breath and asthma [88]<br><br>***Viola tricolor*** **L.**<br>– used in the treatment of skin diseases [108]<br>– used in the treatment of rashes in children [57]<br>– used in the treatment of scabies [112]	
**plants used in rituals**	
Avena sativa **L.**<br>**fruit of the shrub of the *Corylus* L.**<br>– fertility stimulants, common practices of sprinkling oats and hazelnuts, for example, on St. Stephen's Day (December 26), boys would throw oats and hazelnuts at girls after Mass [120]<br><br>***Pisum sativum*** **L.**<br>***Triticum*** *aestivum* **L.**<br>– fertility stimulants, common practices of sprinkling peas and wheat, for example, newlyweds were showered with peas "so they would have as many children as there were grains" and with wheat "so that they would have abundance and plenty of bread" [121]<br><br>***Viburnum opulus*** **L.**<br>– used in wedding rituals [122]	***Carduus*** **L.**<br>***Cirsium arvense*** **(L.) Scop.**<br>***Silybum marianum*** **(L.) Gaertn.**<br>***Onopordum acanthium*** **L.**<br>– during the initiation of a novice into the group of reapers, to test their endurance, their face would be rubbed with thistle [123], or they would be dragged with the largest and sharpest thistle across their bare skin [124]<br><br>***Urtica dioica / Urtica urens***<br>– during the initiation of a novice into the group of reapers, to test their endurance, a nettle would be placed around their Neck [125]
**plants blessed during the year**	
Avena sativa **L.**<br>– blessed with other herbs on the Feast of the Assumption of the Blessed Virgin Mary [126]<br><br>**fruit of the shrub of the *Corylus* L.**<br>– blessed with other herbs on the Feast of the Assumption of the Blessed Virgin Mary [126]<br><br>***Fragaria vesca*** **L.**<br>– blessed in wreaths during the octave of Corpus Christi [55]	***Agrostemma githago*** **L.**<br>– blessed in wreaths during the octave of Corpus Christi [88, 126]<br>– blessed with other herbs on the Feast of the Assumption of the Blessed Virgin Mary [55]<br><br>***Carduus*** **L.**<br>***Cirsium arvense*** **(L.) Scop.**<br>***Silybum marianum*** **(L.) Gaertn.**<br>***Onopordum acanthium*** **L.**<br>– blessed in wreaths during the octave of Corpus Christi

– blessed with other herbs on the Feast of the Assumption of the Blessed Virgin Mary [88]<br><br>**_Hypericum perforatum_ L.**<br>– blessed in wreaths during the octave of Corpus Christi [107]<br>– blessed with other herbs on the Feast of the Assumption of the Blessed Virgin Mary [55]<br><br>**_Phaseolus vulgaris_ L.**<br>– blessed with other herbs on the Feast of the Assumption of the Blessed Virgin Mary [123]<br><br>**_Pisum sativum_ L.**<br>– blessed with other herbs on the Feast of the Assumption of the Blessed Virgin Mary [79, 126]<br><br>**_Triticum aestivum_ L.**<br>– blessed with other herbs on the Feast of the Assumption of the Blessed Virgin Mary [126, 127]<br><br>**_Vaccinium myrtillus_ L.**<br>– blessed with other plants in the palm for Palm Sunday [88]<br><br>**_Viburnum opulus_ L.**<br>– blessed in wreaths during the octave of Corpus Christi [126]<br>– blessed with other herbs on the Feast of the Assumption of the Blessed Virgin Mary [126]<br><br>**_Viola tricolor_ L.**<br>– blessed in wreaths during the octave of Corpus Christi [55]	[128]<br>– blessed with other herbs on the Feast of the Assumption of the Blessed Virgin Mary [127]<br><br>**_Sambucus nigra_ L.**<br>– blessed in wreaths during the octave of Corpus Christi [129]<br>– blessed with other herbs on the Feast of the Assumption of the Blessed Virgin Mary [127]<br><br>**_Urtica dioica_ L. / _Urtica urens_ L.**<br>– blessed in wreaths during the octave of Corpus Christi [107, 126]<br>– blessed with other herbs on the Feast of the Assumption of the Blessed Virgin Mary [109]
**plants used for apotropaic purposes**	
**_Hypericum perforatum_ L.**<br>– carried as a remedy against curses [55]<br><br>**_Pisum sativum_ L.**<br>– pea decoction protected cows from charms [79]	**_Agrostemma githago_ L.**<br>– with the blessed flower of the corncockle, housewives would fumigate “bewitched” cows [130]<br><br>**_Carduus_ L.**<br>**_Cirsium arvense_ (L.) Scop.**<br>**_Silybum marianum_ (L.) Gaertn.**<br>**_Onopordum acanthium_ L.**<br>– it was placed on the threshold to protect against witches [131]<br><br>**_Sambucus nigra_ L.**<br>– twigs of black elder were placed in windows, doors, and farm buildings to protect them from witches [71]<br><br>**_Urtica urens_ L.**<br>– twigs of nettle were inserted into roofs [129], in Windows [132] for protection against witches – spoiled cow's milk that was bewitched by witches was strained through nettle to “repair” it [133]
**plants associated with the devil / used in black magic**	
**_Pisum sativum_ L.**	**_Agrostemma githago_ L.**

– used in black magic – to provoke an argument [134], cast a spell [46], bring death to someone [135]	– attracts lightning [136]<br>– it should not be picked, as this may lead to death by lightning and Possesion [84]<br>– the devil often resides in the corncockle [84]<br><br>***Sambucus nigra*** **L.**<br>– an untouchable skrub [137]<br>– it is forbidden to dig it up [138], cut it [133] without risking unpleasant consequences, such as illness [139], paralysis, the withering of the hand [71], death [133]<br>– the shrub is inhabited by numerous demonic beings: an evil spirit [71], the devil [139]<br><br>***Nicotiana tabacum*** **L.**<br>– has a demonic origin [75]<br>– an attribute of demonic and supernatural beings [37]
